# A rare case of eosinophilic jejunitis: diagnosis and management strategies

**DOI:** 10.1093/jscr/rjae157

**Published:** 2024-03-17

**Authors:** T Y Aaboudech, F Zouaidia, K Znati, Z Bernoussi, A Jahid

**Affiliations:** Pathology Department, Faculty of Medicine and Pharmacy, Ibn Sina Hospital, Mohammed V University, Rabat 10100, Morocco; Pathology Department, Faculty of Medicine and Pharmacy, Ibn Sina Hospital, Mohammed V University, Rabat 10100, Morocco; Pathology Department, Faculty of Medicine and Pharmacy, Ibn Sina Hospital, Mohammed V University, Rabat 10100, Morocco; Pathology Department, Faculty of Medicine and Pharmacy, Ibn Sina Hospital, Mohammed V University, Rabat 10100, Morocco; Pathology Department, Faculty of Medicine and Pharmacy, Ibn Sina Hospital, Mohammed V University, Rabat 10100, Morocco

**Keywords:** eosinophilic gastrointestinal disorders, eosinophilic gastroenteritis, hypereosinophilic syndrome

## Abstract

Eosinophilic gastroenteritis is a rare disease with an unknown cause, which can manifest independently or as part of a hyper-eosinophilic syndrome. The severity of the condition depends on the extent of eosinophilic infiltration and damage to the digestive tract. Diagnosis relies on histological examination, which reveals a significant presence of eosinophilic polymorphonuclear leukocytes in the digestive wall. The authors present a new case of eosinophilic gastroenteritis in a 28-year-old patient who exhibited obstructive symptoms but lacked peripheral eosinophilia. Esophagogastroduodenoscopy showed no abnormalities, but barium transit imaging revealed gastro-duodeno-jejunal dilation upstream of a tight jejunal stenosis. Surgical examination of the affected area confirmed a diffuse and transparietal eosinophilic infiltrate, with no evidence of parasitic or granulomatous lesions. Fortunately, the patient had a swift recovery following surgery. Biopsies conducted at other locations, including the gastric, hepatic, and medullary levels, produced negative results, indicating the localized nature of the condition.

## Introduction

Eosinophilic gastroenteritis (EGE) is a rare condition with an as-yet-unknown etiology [1–3]. It is characterized by the association of nonspecific gastrointestinal symptoms with a massive inflammatory infiltrate of eosinophilic polymorphonuclear cells localized in one or more segments of the digestive tract, without systemic involvement and without any triggering parasite or allergic factors [1]. In this case report, we will discuss the epidemiological, clinical, diagnostic, and therapeutic aspects of EGE through a review of the literature.

## Case presentation

**Figure 1:**
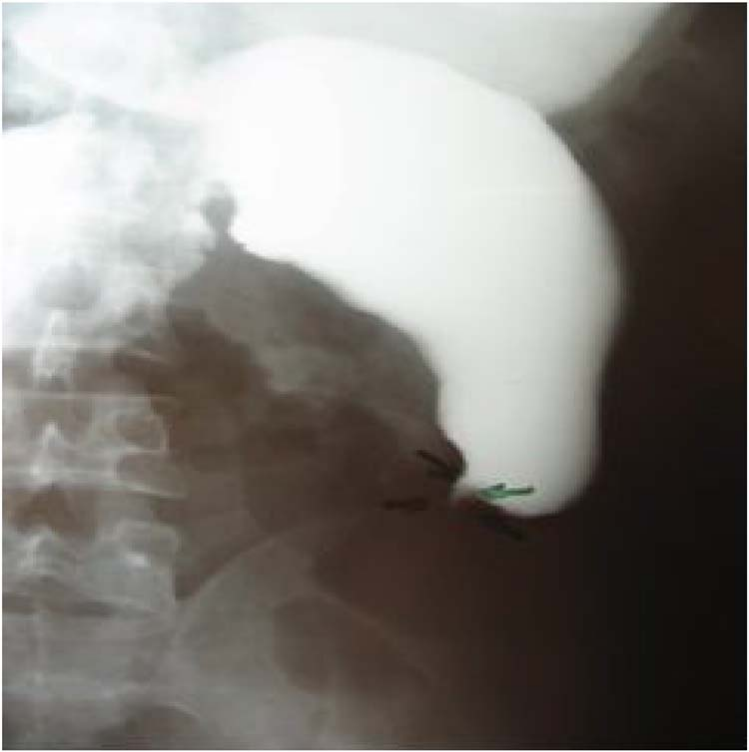
Esogastroduodenal transit showing gastroduodenal dilatation upstream of a tight jejunum stenosis.

A 28-year-old male patient, with no notable medical history, had been experiencing epigastric abdominal pain for a year, followed by early postprandial vomiting without gastrointestinal transit disorders, in the context of unquantified progressive weight loss. Upon admission, the patient was in poor general condition, had no fever, with slightly pale conjunctivae. Abdominal examination revealed epigastric succussion splash with a downward dullness, but no hepatosplenomegaly. Lymph nodes were nonpalpable. The rest of the physical examination showed no particular findings. Laboratory tests showed hypokalemia at 2.96 meq/l and anemia with a hemoglobin level of 10.6 g/dl. Neutrophils and eosinophil counts were normal. Urgent esophagogastroduodenoscopy revealed significant gastric stasis hindering the examination, and an aspiration tube was placed, removing 1500 ml of fluid. After gastric emptying, the endoscopic examination was unremarkable except for a slightly congestive appearance of the antro-fundic mucosa, and systematic biopsies were performed. The histopathological examination showed interstitial antro-fundic lesions without specific or tumoral signs. Esophagogastric-duodenal transit imaging visualized a gastroduodenojejunal dilation upstream of a tight jejunal stenosis, suggestive of a highly probable malignant tumor process (Fig. 1). A stay in the intensive care unit to stabilize and monitor the hemodynamic status followed by surgical intervention was decided.

Intraoperatively, exploration revealed a stenosing lesion of 10 cm from the first jejunal loop with significant dilation upstream. Downstream, there were multiple constrictions with small regional lymph nodes, but no palpable tumor. An extemporaneous examination of a mesenteric lymph node showed no specific or tumoral lesions.

**Figure 2:**
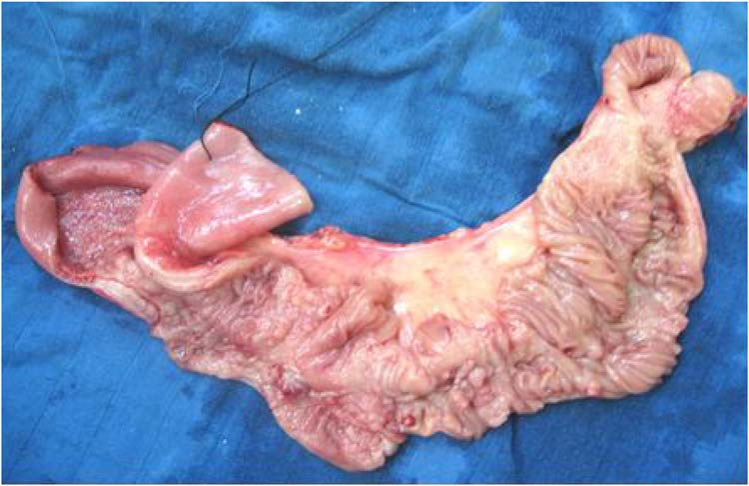
Gross appearance of jejunal loop stenosis.

A 50-cm long jejunal resection, including the entire affected portion, with jejunojejunal anastomosis, was performed. Fresh pathological examination of the surgical specimen noted a congestive segment with thickening of the mesentery containing 15 lymph nodes. Upon opening, the mucosa was extensively ulcerated, with multiple polypoid formations but no fissure or fistulous tracts (Fig. 2).

**Figure 3:**
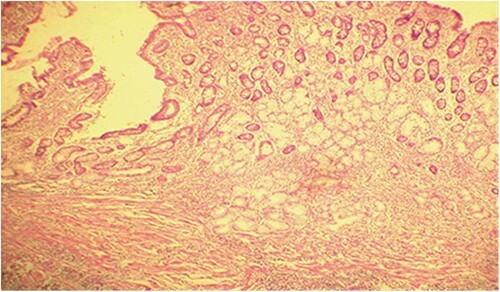
Diffuse infiltration of eosinophilic polymorphonuclear leukocytes affecting all layers of the jejunal wall (hematoxylin–eosin staining, magnification × 100).

**Figure 4:**
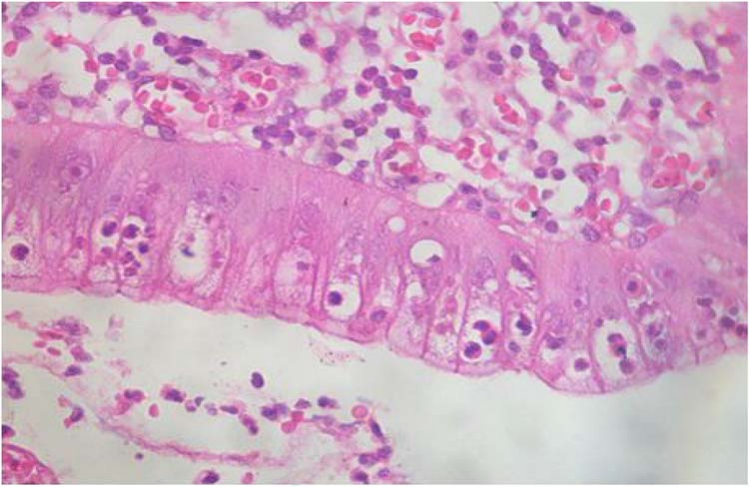
Intraepithelial exocytosis of eosinophilic polymorphonuclear leukocytes (hematoxylin–eosin staining, magnification × 200).

Microscopic analysis of various samples showed a transparietal massive infiltration of eosinophils with over 25% epithelial exocytosis and no granulomatous lesions or parasitic agents (Figs 3 and 4). Lymph nodes sampled from the congestive mesentery were reactive. The diagnosis of eosinophilic enteritis was established. The postoperative course was rapidly favorable, with weight gain. The patient was readmitted, and etiological assessment as well as a search for other locations, including hepatic and osteomedullary biopsies, returned negative results.

## Discussion

EGE is a rare disease. Since it was first described by Kaijer in 1937, ~300 cases have been reported in the English literature [1, 4, 5]. This condition is defined by the association of nonspecific gastrointestinal symptoms with a massive inflammatory infiltrate of eosinophilic polymorphonuclear cells localized in one or more segments of the digestive tract, without systemic involvement and without any triggering parasitic or allergic factors [4]. Infiltration of the digestive tract wall by eosinophilic polymorphonuclear leukocytes can manifest as isolated gastroenteritis or be part of the essential hyperesinophilic syndrome. Prolonged eosinophilia and visceral lesions, especially cardiac, pulmonary, and neurological ones, characterize this syndrome [6]. The majority of cases have been reported in Caucasians, with a slight male predominance. The most affected age group is between the second and fifth decades as was the case with our patient. A history of allergy, although present in half of the cases, is not mandatory for diagnosis [1, 4]. Our patient did not report any personal or family history of atopy or food or drug allergies.

Classically, the clinical presentation of EGE varies depending on the depth of infiltration into the digestive wall. According to the classification established by Klein in 1970 [7], when the infiltrate predominates in the mucosa, clinical signs are primarily nausea, vomiting, abdominal pain, diarrhea with malabsorption, and exudative enteropathy. When the muscular layer is affected, obstructive syndromes often take the forefront. Finally, predominant serosal involvement is a rarer form, which is associated with ascites rich in eosinophils and occasionally pleural effusion. The clinical picture presented by our patient (abdominal pain, immediate vomiting, tight jejunal stenoses, and ascites) constitutes the anatomical lesion substrate of transparietal involvement of the jejunal loop. Currently, clinical manifestations also depend on the height of histological infiltration in the digestive tube [1, 8]. Thus, isolated esophageal involvement, where dysphagia is the main clinical sign, is rarely symptomatic and often concomitant with another more symptomatic location (stomach, small intestine, and serosa), and its frequency is probably underestimated [9]. Gastric involvement is one of the most common (40%–70% of cases), mainly affecting the antrum and resulting in nausea, vomiting, abdominal pain, and weight loss. Sometimes, it presents as gastric or pyloric stenosis, which is diagnosed intraoperatively. Small bowel involvement is relatively common (10%–25% of cases) and predominantly affects the duodenum and ileum, which is accompanied mainly by diarrhea with malabsorption and exudative enteropathy. Jejunal involvement is rarely observed and is often complicated by an acute surgical abdomen as was the case in our patient. A few cases of intestinal perforations have been reported. By contrast, colonic locations of EGE are rarer [10]. These are mostly ileocolic forms that can pose diagnostic challenges with Crohn’s disease. Peritoneal involvement, also infrequent (<10% of cases), results in ascites, which is often transient. Preceding bloating is nonspecific. This form is generally associated with severe peripheral eosinophilia [11]. During laparoscopy, the peritoneum appears to be hypervascularized, possibly suggesting peritoneal carcinomatosis. Pancreatic involvement is exceptionally rare in EGE and is characterized by pseudotumoral forms involving the duodenum or gastric antrum, which is inconsistently associated with peripheral eosinophilia and a history of atopy.

Biologically, EGE is characterized by blood eosinophilia present in half of the cases (its level is particularly high in serous forms of the disease), with the presence of an inflammatory syndrome (25% of cases) and an increase in total IgE levels (50% of cases) [12]. In our case, there was neither peripheral eosinophilia nor elevated inflammatory markers. IgE levels were not measured.

Endoscopic examination shows nonspecific findings; most often, there is erythematous, ulcerative and nodular mucosa, and sometimes it resembles peptic ulcers. Hence, the need for multiple and fairly deep biopsies to avoid unnecessary surgical procedures [1, 6, 13]. The preoperative gastroscopy performed in our patient found erythematous gastric mucosa with mild chronic antro-fundic lesions that were nonspecific on histological examination of gastric biopsies. Ultrasound can be of great interest as it shows generalized thickening of the intestinal wall, allowing for the evaluation of therapeutic response by comparing lesion thickness. Additionally, ascites discovery permits cytological analysis. Barium studies indicate wall and fold thickening, narrowing the lumen. Computed tomography lacks added insight, often showing nonspecific fold thickening [1, 13]. Suspicious radiological findings during upper gastrointestinal contrast imaging prompted immediate surgical exploration due to tight stenoses resembling a tumor. The definitive diagnosis relies on the histological examination of biopsy samples, revealing significant infiltration of the digestive wall by eosinophilic polymorphonuclear leukocytes with intraepithelial exocytosis exceeding 20% per field [1]. This is accompanied by epithelial hyperplasia with crypt elongation. The interstitium generally shows significant exudate with microabscesses. The search for granulomas or parasitic agents is negative. Differential diagnosis [8] should consider parasitic- and drug-related conditions. The effect of corticosteroid therapy is spectacular, both clinically, biologically, and radiologically [3–5, 14]. The initial dose is 20–40 mg/day of prednisone for 15 days, followed by 5–10 mg/day for maintenance treatment. However, this effectiveness is not constant, and the duration of treatment is not well codified [15]. Some acute forms may require surgical resection for diagnostic and therapeutic purposes as was the case with our patient. Other subacute or chronic forms, which more often express as a malabsorption syndrome with predominant mucosal involvement, regress with topical corticosteroid treatment (budesonide) at a dose of 9 mg/day [3, 4].

## Conclusion

This finding aligns with most reported cases of EGE in terms of symptoms and progression. Yet, strict jejunal involvement is rare, typically lacking peripheral eosinophilia. Clinical signs and imaging results often vary. Surgical confirmation is advised due to the transmural nature, challenging endoscopic access. Despite occasional chronicity with steroid treatment, the prognosis generally remains favorable.

## Author contributions

T.Y.A. conceptualized and designed the study, collected and analyzed the patient data, and wrote the paper. F.Z. assisted in data collection, conducted literature review, contributed to the interpretation of the findings, and critically revised the manuscript for important intellectual content. K.Z. Provided clinical expertise and guidance, reviewed and verified the accuracy of patient-related information, and contributed to the final editing and formatting of the manuscript. Z.B. contributed data or analysis tools and performed the analysis. A.J. supervised the entire research process, reviewed and approved the study design and methodology, and provided critical revisions and intellectual input to ensure the scientific rigor and integrity of the case report.

## Conflict of interest statement

None declared.

## Funding

None declared.

## Ethical approval

We further confirm that any aspect of the work covered in this manuscript that has involved human patients has been conducted with the ethical approval of all relevant bodies and that such approvals are acknowledged within the manuscript.

## References

[1] Amit M , Chijioke E, David G, et al. Eosinophilic gastroenteritis: review of a rare and treatable disease of the gastrointestinal tract. Case Rep Gastroenterol 2013;7:293–8. 23904840 10.1159/000354147 PMC3728613

[2] Tagore S , Prashanth R, Sowjanya YK, et al. Eosinophilic gastroenteritis: diagnosis and clinical perspectives. Clin Exp Gastroenterol 2019;12:239–53. 10.2147/CEG.S173130. 31239747 PMC6556468

[3] Uenishi T , Sakata C, Tanaka C, et al. Eosinophilic enteritis presenting as acute intestinal obstruction: a case report and review of the literature. Dig Surg 2003;20:326–9. 10.1159/000071759. 12806199

[4] Ingle SB , Hinge CR. Eosinophilic gastroenteritis: an unusual type of gastroenteritis. World J Gastroenterol: WJG 2013;19:5061. 23964139 10.3748/wjg.v19.i31.5061 PMC3746377

[5] Siewert E , Lammert F, Koppitz P, et al. Eosinophilic gastroenteritis with severe protein-losing enteropathy: successful treatment with budesonide. Dig Liver Dis 2006;38:55–9. 10.1016/j.dld.2005.06.013. 16326154

[6] Yasuhiro F , Kojiro T, Akira H, et al. Endoscopic findings of gastric lesions in patients with eosinophilic gastrointestinal disorders. Endosc Int Open 2020;08:E1817–25. 10.1055/a-1268-7312. PMC7676993 33269315

[7] Shefali A , Sandeep V, Sangeeta R, et al. Eosinophilic ascites: a diagnostic and therapeutic challenge. World J Gastroint Surg 2016;8:656. 10.4240/wjgs.v8.i9.656. PMC5037340 27721930

[8] Álamo Martínez JM , Ibáñez Delgado F, Galindo Galindo A, et al. Aspectos quirúrgicos de la enteritis eosinofílica. Rev Esp Enferm Dig 2004;96:279–83. 15259143 10.4321/s1130-01082004000400008

[9] Zink David A , Mitual A, Souheil G, et al. Familial dysphagia and eosinophilia. Gastrointest Endosc 2007;65:330–4. 17258999 10.1016/j.gie.2006.07.021

[10] Yalon M , Amawi T, Ali D, Kelm ZS, et al. Eosinophilic disorders of the gastrointestinal tract and associated abdominal viscera: imaging findings and diagnosis. Radiographics 2022;42:1081–102. 35749291 10.1148/rg.220004

[11] Mazokopakis E , Vrentzos G, Spanakis E, et al. A case of eosinophilic gastroenteritis with severe peripheral eosinophilia. Mil Med 2006;171:331–2. 10.7205/MILMED.171.4.331. 16673749

[12] Khan S . Eosinophilic gastroenteritis. Baillieres Best Pract Res Clin Gastroenterol 2005;19:177–98. 10.1016/j.bpg.2005.01.009. 15833687

[13] Naoya K , Yasuharu Y, Taro Y, et al. Multiple ulcerative lesions of the stomach: a rare case of eosinophilic gastroenteritis. Gastrointest Endosc 2002;56:762–4. 12397296 10.1067/mge.2002.129216

[14] Robert ME . Inflammatory Disorders of the Small Intestine in Surgical Pathology of the GI Tract, Liver, Biliary Tract, and Pancreas (Second Edition). Elsevier, 2009.

[15] Alobid I , Backe V, Canevari F, et al. Oral corticosteroids for patients with eosinophilic diseases: an expert panel view on use, overuse, and strategies to reduce use. Metabolic acidosis in children: a literature review. EMJ 2023;8:69–79. 10.33590/emj/10303904.

